# Green synthesis of silver nanoparticles with algae and the importance of capping agents in the process

**DOI:** 10.1186/s43141-021-00228-w

**Published:** 2021-08-24

**Authors:** Deeksha Chugh, V. S. Viswamalya, Bannhi Das

**Affiliations:** Department of Biotechnology, Mount Carmel College, Autonomous, Bangalore, 560052 India

**Keywords:** Algal synthesis, Capping agent, Green synthesis, Silver nanoparticles, Transmission electron microscope

## Abstract

**Background:**

Nanoparticle synthesis is a very interesting area of research currently due to the wide applications of nanoparticles. The nanoparticles have a diameter ranging between 1 and 100 nm and they are used in different fields like electronics, pharmaceuticals, cosmetics, biotechnology, medicines, etc.

**Main body of the abstract:**

Nanoparticles have gained the interest of researchers due to their large surface-to-volume ratio and their capability to interact effectively with other particles. Several different methods can be used for the production of silver nanoparticles (AgNPs) including chemical, physical, and biological. Out of all the methods, the biological method is considered the cleanest and safest as no toxic chemicals are used in the process. The biological method includes the use of bacteria, fungi, algae, and plant extract for the synthesis. Algal synthesis of AgNPs is especially interesting because of the high capacity of the algae to take in metals and reduce metal ions. Algae is a widely distributed organism and its availability is abundant; an added advantage is their growth under laboratory conditions. These organisms can help in large-scale production at a low cost.

**Short conclusion:**

This review article explains the different factors that should be considered for the effective synthesis of AgNPs using algae. Capping agents also affect the stability of nanoparticles. It also sheds light on the importance of capping agents in the synthesis of AgNPs. Alga-mediated synthesis of AgNPs along with the use of different capping agents can help in modulating the stability and size of the nanoparticles, thereby improving its cost-effectiveness and environment-friendly production.

**Graphical abstract:**

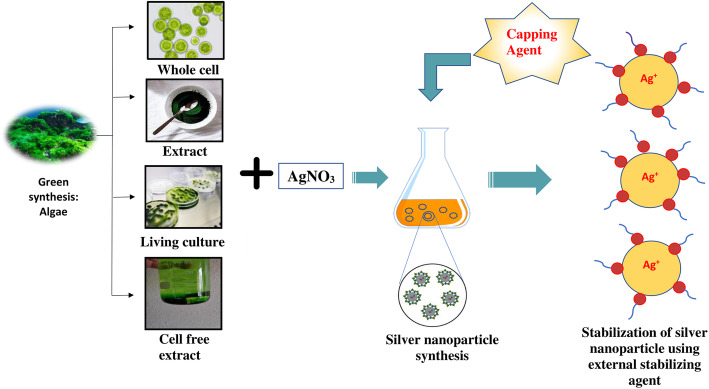

## Background

There are several different microorganisms like bacteria, molds, fungi, etc. that are known to cause infection in humans and other living organisms. To combat these infections, several antibiotic and antimicrobial compounds have been discovered. But, slowly with time, micro-organisms started exhibiting resistance against most of the antibiotics thereby creating a problem in treating these microbial diseases [1]. Researchers have been focusing on alternative therapies or methods that can overcome this antibiotic resistance along with cost-effectiveness [2]. Nanoparticles (NP) are being considered as a good alternative candidate in many cases.

For over the last 2000 years, silver has been known for its medicinal properties as they possess broad-spectrum antimicrobial activity. There are several different modes by which silver shows inhibitory action against microorganisms. Silver-based products have a lesser tendency to induce antimicrobial resistance and are cost-efficient [2, 3]. Nanotechnology is a relatively new discipline and one of the fast-growing areas of science. It is of great interest to researchers due to its wide application in industries like food, cosmetics, medicines, agriculture, chemical, and pharmaceuticals. Nanotechnology is concerned with the synthesis, design, and exploitation of the structure of a particle ranging in size from roughly 1 to 100 nm. It describes how different parameters like origin, structure, and chemical properties can affect the functionality of nanomachinery of cells, and also examine the relation between nanomaterials and nano-biosystems [4, 5]. The characteristics of nanoparticles depend on several parameters like shape, size, nature of nanoparticles, and surroundings [4–6].

There are several distinct techniques by which silver nanoparticles (AgNP) can be synthesized. They can be produced using physical, chemical, and biological methods. By and large, two approaches are associated with the formation of silver nanoparticles, either from the “top to bottom” approach or a “bottom to top “ approach. As the name suggests, in the “bottom to top” approach, smaller atoms of molecules assemble themselves to form nanoparticles. On the other hand, the “top to bottom approach” is the opposite. The synthesis of nanoparticles takes place in this process by breaking down the bulk material into fine size particles using numerous lithographic techniques such as grinding, milling, sputtering, and thermal/laser ablation [5]. The physical method of synthesis involves several different techniques like ball milling, thermal evaporation, ultra-thin films, lithographic techniques, plasma arcing, and diffusion flame synthesis [7]. Chemical synthesis is one of the most common methods used to produce silver nanoparticles. Several different chemicals and reagents are used to reduce the silver ions and stabilize the nanoparticles [7–9]. Some of the methods involved in this process are chemical solution deposition, catalytic route, sol-gel process, Langmuir-Blodgett method, wet chemical method, etc. [7]. But one of the biggest drawbacks of this is that the chemicals used for the synthesis are toxic and can be harmful to human health and the environment. So, another approach that gained the attention of several researchers is the use of biological agents or organisms for the synthesis of nanoparticles. This is known as biogenic synthesis and this method has less toxicity, high stability, and better physiochemical characteristics.

**Fig. 1:**
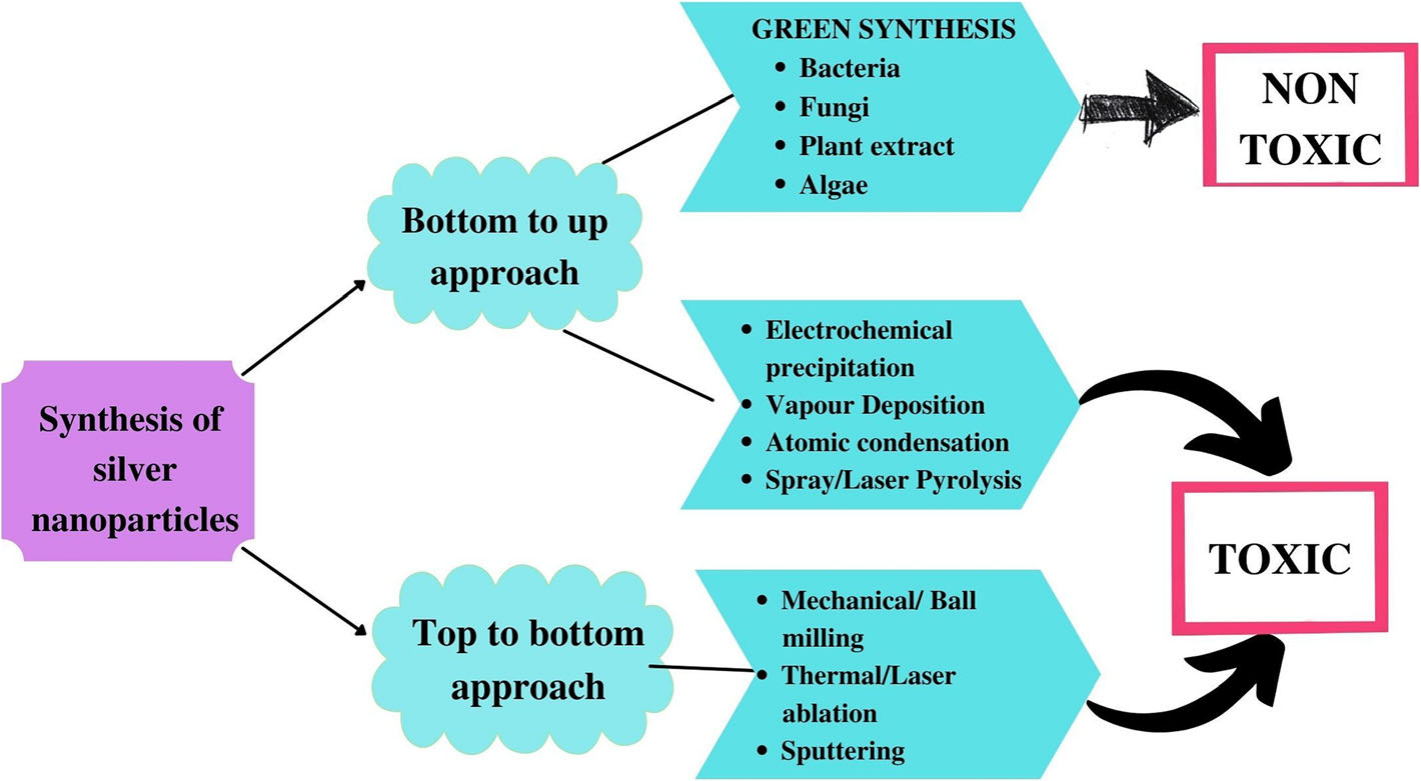
Different approaches that can be used for the synthesis of silver nanoparticles

Biogenic synthesis of nanoparticles can be carried out by the use of organisms like fungi, algae, bacteria, plants, and their metabolites, which serve as reducing and stabilizing agents [4–6, 8]. All the different approaches that can be used to synthesize nanoparticles are depicted in Fig. 1.

The antimicrobial activity of AgNPs is due to their combined effects of binding of Ag ions to the cell walls, inactivation of the membrane-associated enzymes, accumulation within the cells, interference with the essential biomolecules of the bacterial cells, denaturation of the cell envelope, and the formation of reactive oxygen species. All of these events are detrimental for the bacterial cells [2, 3, 9, 10]. It has been experimentally proven that suspension of AgNPs shows antimicrobial activity against reference and hospital-isolated bacterial strains of *Pseudomonas aeruginosa*, which were resistant to the multiple antibiotics [11]. Silver nanoparticles exhibit effective antimicrobial properties compared to other nanoparticles because of their extremely large surface area, which offers improved interaction with microorganisms [12]. There are multiple studies that demonstrate that Ag-NPs are effective even at very low concentrations against MRSA (methicillin-resistant *Staphylococcus aureus*) strains. AgNPs cause a disruption of their cell wall. It can act as an effective substitute for the treatment of medical device-associated infections caused by drug-resistant strains of bacteria [3, 13]. Moreover, AgNPs are also used in the electronics and electric field due to their high stability, conductivity, and high performance. AgNPs are also in demand in the textile industry and the food and beverages industry for food storage and packing purposes [14]. Recently, they have been successfully applied for the detection and treatment of cancer also [5]. The projected market value of nanoparticles for their antimicrobial role has increased from $0.79 billion in 2014 to $2.54 billion in 2022 [15]. In this article, we have focused on the different methods used to date for the green synthesis of silver nanoparticles with a special emphasis on algae. We have attempted to bring in the factors that can help in modulating the process of synthesis. An in-depth discussion is expected to provide insight into the algal production of AgNP.

## Applications of silver nanoparticles

**Fig. 2:**
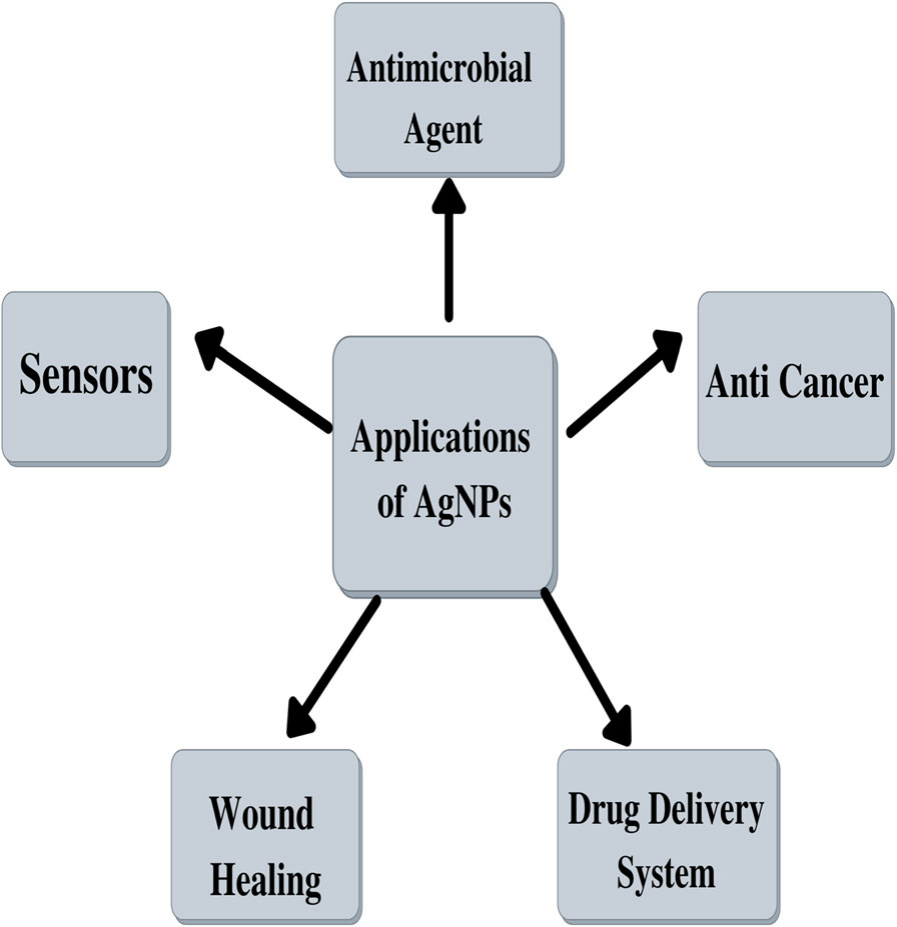
Applications of silver nanoparticles

AgNP has unique physical, chemical, and electrical properties, and because of this, they have several applications in different fields like medicines, optics, water treatment, food industry, etc. (Fig. 2). They can be integrated into composite fibres, cryogenic superconducting materials, and electronic components. Due to its wide range of applications, there is a requirement for advancement in a cost-effective method for synthesis. AgNPs are used extensively in the field of medicine due to the spread of infectious diseases and the emergence of multidrug resistance in pathogens which is rendering the existing antibiotics ineffective [10, 16]. AgNPs are being exploited in the pharmaceutical industry due to their low toxicity in the cells of humans and their stability at various temperatures. Due to these extensive applications of AgNPs, their synthesis has gained a lot of attention from researchers worldwide [17, 18].

## Antimicrobial agent

AgNPs are widely used as antimicrobial agents as they have the potential to overcome antibiotic resistance. They effectively work against gram-positive as well as gram-negative bacteria. It has been reported that AgNP interacts with the bacterial cell wall, penetrates it, and causes serious disturbance in cell functioning which causes cell death [3, 9, 19]. Several different physicochemical parameters affect the antimicrobial properties of AgNPs and they include size, shape, concentration, surface charge, and colloidal state [20]. These physicochemical properties are mostly dependent on the method used for the synthesis of the nanoparticle. The antimicrobial activity exhibited by AgNPs synthesized by different algae is listed in Table 1.

**Table 1:** Antimicrobial activity of algae-synthesized silver nanoparticles

Algae	Size of NP/shape	Volume of NP used	Antimicrobial activity (zone of inhibition)	Reference
***Padina tetrastromatica***	14 nm/spherical	30 μl, 60 μl, 90 μl	*Bacillus* sp., *Pseudomonas* sp., *Bacillus subtilis*, *Klebsiella planticola*	[21]
***Sargassum polycystum***	5–7 nm/spherical	100 μl	*Pseudomonas aeruginosa*, *Klebsiella*, *Pneumoniae*, *Escherichia coli*, *Staphylococcus aureus*	[22]
***Gracilaria corticata***	18–46 nm/spherical	30 ml	*C. albicans* *C. glabara*	[23]
***Gelidiella acerosa***	22 nm/spherical	50 μl	*Humicola insolens*, *Fusarium dimerum*, *Mucor indicus*, *Trichoderma reesei*	[23]
***Enteromorpha flexuosa***	2–32 nm/circular		*B. subtilis*, *B. pumulis*, *E. faecalis*, *S. aureus*, *S. epidermidis*, *E. coli*, *S. cerevisiae*, *C. albicans*	[24]
***Sargassum wightii***	8–27 nm/spherical	30 μl, 60 μl, 90 μl	*Staphylococcus aureus*, *Bacillus rhizoids*, *E. coli*, *Pseudomonas aeruginosa*	[25]
***Turbinaria conoides***	2–17 nm/spherical	50 ml	*Salmonella*, *E. coli*, *S. liquefaciens*, *A. hydrophila*	[26]
***Ulva fasciata***	50 nm/spherical	20 μl	*C. albicans*, *C. glabrata*	[27]

Sondi et al. in an experiment showed that AgNP gets attached to the cell wall of *Escherichia coli* and form holes on the membrane which ultimately leads to cell death [28]. They also demonstrated that the size of silver nanoparticles influences antimicrobial activity; smaller particles were more potent than the larger-size particles [28]. AgNPs show both bactericidal and bacteriostatic activity against biofilm-forming organisms. Raffi et al. reported that AgNP at a concentration of 60μg/ml or more is effective in killing *E. coli* establishing its bactericidal ability [29].

## Anti-cancer agents

Nowadays, AgNPs are widely used as a therapeutic agent, in the diagnosis and treatment of cancer. This is because the toxicity of silver nanoparticles towards cancerous cells is more as compared to the bulk materials. It has been observed that AgNPs acts as an antitumour agent by suppressing tumour cell progression. The probable reason for this can be the inhibitory actions of nanoparticles in several signalling cascades which are required for the development and pathogenesis of cancer. Interestingly, there is no lethal effect of nanoparticles on normal cells [30]. Promising anticancer effects of silver nanoparticles are recently being investigated in different human cancer cell lines, such as endothelial cells, IMR-90 lung fibroblasts, U251 glioblastoma cells, and MDA-MB-231 and MCF-7 breast cancer cells [9, 31–33]. Tumour cells require continuous nutrition and oxygen for their growth and this required an intense network of blood vessels that are formed by the process of angiogenesis. AgNP was reported to block the process of angiogenesis thereby suppressing the growth of the tumour. AgNPs synthesized using *Ecklonia cava* extract were found to be effective against Human cervical cells (HeLa cells) with an IC50 value around 59μg/ml [34, 35].

## Drug delivery system

AgNPs are also being used for targeted drug delivery now a day. Its effectiveness in delivering the anti-cancer drugs to the tumour tissue has already been observed. The size of the nanoparticles makes it easy for them to penetrate the tissue and ensure effective drug delivery [36]. The use of AgNPs for delivering anti-cancer drugs is of great interest to researchers because of the cytotoxic effect shown by the convention methods of drug delivery [36, 37]. The conventional methods cause the death of the normal or healthy cells along with the tumour cells [37].

## Wound healing

Wound healing is a complicated and time-consuming process as several different cell lineages are involved in this process. There are several extrinsic and intrinsic factors that affect the rate of healing including age, size and depth of the wound, medication, nutritive status, etc. Wound management is very important otherwise there are increased chances of infection at the site of injury which can lead to several complications. A perfect wound dressing maintains a moist environment around the wound, ensures protection against microorganisms, and takes off excess fluids or dead cells [38]. Conventionally, 6 different types of drugs are used for wound management and healing, which include antiseptics, tropical antibiotics, granulation tissue suppressing agents, herbal therapeutics, enzyme treatment, and some other tropical agents (vitamin E, scarlet oil). But there are several limitations or drawbacks related to conventional medication therapy, like narrow antimicrobial spectrum, skin irritation, allergies and cytotoxic effect on body cells, etc. AgNPs can be a perfect alternative for conventional medicines as they have low toxicity to the system, effective against pathogens that are multidrug-resistant, and they are not responsible for causing drug resistance due to their multilevel antimicrobial effect [39]. Another advantage of using silver nanoparticles is that it reduces the secretion of cytokines which in turn, decreases the infiltration of mast cells, and therefore, they act as anti-inflammatory agents [40].

## Sensors

Environmental monitoring is the process of accessing the environmental condition to check the concentration of pollutants in the surrounding. Several toxic metals can be present in the environment like mercury, cadmium, lead, copper, nickel, chromium, etc. Due to recent advancements in nanotechnology, it has been found that silver nanoparticles can be efficiently used in optical sensing for the detection of heavy and toxic metals [9, 41] (Table 2). Vasileva et al. demonstrated that starch-coated AgNPs of size 15.4 ± 3.9 nm in the presence of HNO_3_ can be effectively used for the quantification of Hg^2+^ [42]. The principle behind Hg^2+^ sensing is that the absorbance strength optical response of silver nanoparticles depends on the concentration of Hg^2+^ in the solution. The most relevant results were obtained when the Hg^2+^ concentration was in the range of 0.9–12.5 μg L^−1^ and 25–500 μg L^−1^ [42]. Tashkhourian et al. demonstrated the use of chitosan-capped silver nanoparticles (Chit-AgNPs) for the detection of Fe^3+^ ions [43]. The surface plasmon resonance band of silver nanoparticles disappears at a higher concentration of Fe^3^. It is a highly sensitive method of detection and can detect concentrations as low as 0.53μM [43].

**Table 2:** List of silver nanoparticles used for detecting different pollutants

Type of Ag nanoparticle	Pollutant detected	Detectable concentration	Reference
**Riboflavin-stabilized AgNPs**	Hg^2+^	5 nm	[44]
**1-Dodecanethiol (C12h25sh)-capped silver nanoprisms**	Hg (Ii)	10–500 nm	[45]
**Starch-coated AgNps**	Hg^2+^	0.9–12.5 μg L^−1^ & 25–500 μg L^−1^	[42]
**2-Aminopyrimidine-4,6-diol-capped silver nanoparticles (Apd-AgNps)**	Hg^2+^	0–65 μm	[46]
**Citrate (Cit) Andl-cysteine (l-Cys)-capped AgNPs**	Hg^2+^	1–10 Ppm	[47]
**Glutathione-stabilized silver nanoparticles**	Ni^2+^	> 25 nm	[48]
**Casein peptide-capped AgNPs**	Cu^2+^	0.08–1.44 μm	[49]
**Chitosan-capped silver nanoparticles (Chit-AgNPs)**	Fe^3+^	> 0.53 μm	[43]
**Starch-coated silver nanoparticles**	Fe^3+^	0.7–7 mg/l	[50]
**l-Tyrosine-stabilized silver nanoparticles**	Mn (II)	1–10 μm	[51]
**Tartaric acid-capped silver nanoparticles**	Cr (III ) & Cr (VI)	5–100 μg/l Cr (III) & 10–100 μg/l Cr (VI)	[52]

## Solar cell

One of the recent applications of AgNPs is in the dye-sensitized solar cell as they induce surface plasmon resonance which thereby increases the absorption coefficient of dye [53]. Ihara et al. discovered that the efficiency of the dye-sensitized solar cell increased from 1.5 to 2.5% in the presence of polymer-modified AgNPs. Here, the nanoparticles improved the photoelectric conversion efficiency of the solar cell [54].

## Different methods of nanoparticle synthesis

In light of the wide applications of AgNPs, their synthesis becomes a very crucial factor. Three different approaches can be used for the synthesis of nanoparticles, namely physical, chemical, and biological or green synthesis. Each of these methods can be further classified based on the type of chemical/ physical/ biological component being used in the process.

### Physical methods

#### Vapour condensation method

Vaporization and Condensation are two different steps that are involved in this process and are done at atmospheric pressure using a tube furnace. A heat source is used for the process of vaporization and then these vapours are quickly condensed, resulting in nanoparticle synthesis [55]. However, there are some disadvantages such as high energy usage and time consumption to increase the ambient temperature across the source material and to attain thermal stabilit y[56].

#### Arc discharge method

It is another method that can be used for the physical synthesis of nanoparticles. In this method, no surfactants or stabilizers are used and the direct-current arc voltage is applied across two graphite electrodes that are immersed in an inert gas such as He, Ar, or Ne [55]**.** In a study conducted by Tran et al., AgNPs were synthesized in deionized water with no surfactants using the arc discharge method. They used silver wires of 1-mm diameter as electrodes. The results showed that AgNPs synthesized using this method were 10 nm in diameter [56].

#### Laser ablation method

In the laser ablation method, AgNPs can be synthesized in the solution phase with laser ablation on metallic bulk silver salt [55]. It has been shown by Pyatenko et al. that 2–5 nm of spherical silver nanoparticles can be synthesized in pure water by irradiating the Ag target with the laser beam of wavelength 532 nm. The wavelength of the laser beam and the ablation time are the most important factors that determine the size of synthesized nanoparticles [57].

### Chemical Method

#### Chemical reduction method

This is the most commonly used chemical method that involves the use of a reducing agent and a stabilizing agent/capping. Some of the common reducing agents used for AgNP synthesis are sodium citrate, ascorbate, sodium borohydride (NaBH_4_), elemental hydrogen, polyol process, N, N-dimethylformamide (DMF), and poly (ethylene glycol) block copolymers. The reducing agent reduces Ag^+^ ions to Ag metal after which they agglomerate into clusters. Cluster formation can be prevented using stabilizing agents/capping agents [9, 58, 59]. Some of the most commonly used capping agents for the chemical reduction method are citrate, polyvinylpyrrolidone (PVP), cetyltrimethylammonium bromide (CTAB), polyvinyl alcohol (PVA), trisodium citrate, and polyethylene glycol (PEG) [58, 59].

#### Microemulsion method

This approach includes the use of a mixture of water, surfactant, and oil. Sometimes a co-surfactant is also used along with the other components in this process. The commonly used surfactants are anionic surfactants like bis(2-ethylhexyl) sulfosuccinate, sodium dodecylbenzene sulfonate, and lauryl sodium sulphate and cationic surfactants like CTAB, PVP, and non-ionic surfactants like Triton X-100, etc. The surfactant molecules are surrounded by water droplets, which are like a silver nanoparticle synthesis micro reactor [60].

#### Polyol process

This process includes the reduction of silver nitrate (AgNO_3_) at 160 °C with ethylene glycol. PVP acts as a capping agent. The reduction of AgNO_3_ with ethylene glycol and PVP produces monodisperse Ag nanocubes [61].

#### Tollens’ method

Tollens’ reagent and reducing sugars can also be used for the synthesis of AgNPs. Synthesis with these reagents can be carried out at room temperature and the reducing sugars act both as a reducing agent for the Ag^+^ ions and a stabilizing agent over the AgNP surface producing stable anti-bacterial Ag-NPs [62].

## Green synthesis

Green synthesis is a comparatively more eco-friendly approach for the synthesis of metal nanoparticles than the physical and chemical methods. It involves the use of biological agents such as bacteria, fungi, algae, and plant exacts for the synthesi s[7, 8, 13, 63].. There are several studies that showed the use of natural polymers like chitosan, soluble starch, polypeptide, heparin, and hyaluronan as capping and reducing agents during the green synthesis of nanoparticles [64]. The process of green synthesis varies based on the type of organism used or the form in which the organisms are used. Some of the synthesis procedure involves live microbial culture, whereas some involve the use of cell extracts or purified biomolecules from them.

## Microwave-assisted synthesis

This method of synthesis involves the use of microwave heating rather than conventional heating during the process of synthesis. The use of a microwave fastens the reaction and ensures uniform heating. This method aids in the synthesis of silver nanoparticles without agglomeration and reduced energy consumption [58]. X. Zhao et al. synthesized AgNPs using microwave irradiation [64]. Alginate was used as a capping and stabilizing agent for the production. This method does not involve any chemical agent as alginate is a naturally occurring carbohydrate that is isolated from sea algae [64].

## Biological method

Biological methods involve the use of plant extracts and microorganisms. The green synthesis by plants involves only the aqueous solution of the Ag^+^ ions and the plant extracts because plants have the properties of detoxification, reduction, and accumulation of metals. Plant extracts contain compounds like polysaccharides, flavonoids, alkaloids, enzymes, polymers, and proteins as reducing agents and even some of them act as capping agents [12, 13, 65]. Different plant extracts are used to synthesize AgNPs from their leaves, roots, stems, and fruits. Plant extracts of *Azadirachta indica*, *Abutilon indicum*, *Coffea arabica*, *Jatropha curcas*, *Ocimum sanctum*, *Emblica officinalis*, *Aloe vera*, and many more are already being used for AgNP synthesis. The size of the AgNPs synthesized using plant extracts depends on the concentration of the plant extract or the AgNO_3_ which is the source [14]. Microorganisms like bacteria, fungi, algae, and yeast are employed in the green synthesis of AgNPs.

It has been observed that nanoparticles synthesized by biological methods are capped with biomolecules like alkaloids, phenolic compounds, terpenoids, enzymes, co-enzymes, proteins, polysaccharides, lipids, etc. [63]. These biomolecules are present in plants, algae, fungi, bacteria, and yeast. Flavonoids, terpenoids, alkaloids are present in plants. Algal biomolecules are mostly proteins, polyunsaturated fatty acids, vitamins, tocopherols, polyphenols, carotenoids, etc. [63]. However, bacterial and fungal biomolecules that assist in nanoparticle synthesis are mostly Proteins and enzymes [66–69]. Capping agents have multiple roles: prevent agglomeration, reduce toxicity, and increase stability [63].

The green synthesis using microbes is gaining special attention due to the easy handling of microbes, sustainability, and its economic benefit over the other methods [8].

## Problems associated with the physical and chemical methods of synthesis

The different physical and chemical methods mentioned above are extremely expensive and not environment-friendly. The chemical methods use toxic and hazardous chemicals that pose an environmental and biological risk. The physical methods involve high energy requirements [70]. The physical and photochemical methods require very high temperature, vacuum conditions, and costly equipment, to prepare nanoparticles [71]. Physical and chemical methods are also associated with low production rate, structural particle deformation, and particle growth inhibition during the synthesis of nanoparticles.

The synthesized nanoparticles have a wide array of applications, hence their economic and environmentally safe production is very important [3]. Hence, there is currently an increasing need to improve the sustainable preparation of nanoparticles that minimize the use of hazardous organic chemical compounds [70]. The focus has thus moved from physical and chemical processes to 'green' chemistry and bioprocesses for this synthesis [72].

## Biogenic synthesis of silver nanoparticles using algae

Algae are a group of autotropic organisms with economic and ecological importance. They are single or multicellular organisms that live in different habitats, such as freshwater, marine water, or damp rock surfaces. The two different categories of algae are microalgae (microscopic) and macroalgae (macroscopic). They play a vital role in applications such as medicines, pharmacy, forestry, aquaculture, and cosmetics. They are an important source of several commercial products including natural dyes and biofuel [73].

The branch of nanoscience that deals with the synthesis of nanoparticles using algae is termed “Phyconanotechnology”. It is a comparatively new branch of nanoscience. Algae are used for the synthesis of nanoparticles as they have a high potential to accumulate metal, are easy to handle and cultivate, can grow at low temperatures, and are less toxic to the environment [74]. *Chlorophyceae*, *Phaeophyceae*, *Cyanophyceae*, and *Rhodophyceae* are the most common types of algae that are used for the synthesis of silver nanoparticles [73]. Table 3 exhibits AgNPs synthesized by different species of algae, along with the size and shape of the NPs synthesized.

**Table 3:** Properties of silver nanoparticles synthesized using various classes of potential algae

Algae	***Species***	Synthesis	Size of Np (Nm)	Shape	Temp.	Reference
**Cyanobacteria**	*Microcoleus*	Extracellular	44–79	Spherical	RT	[75]
	*Phormidium willei*	Extracellular	100–200	Spherical	25	[76]
	*Plectonema boryanum*	Intracellular & Extracellular	1–15,1–40, 5–200	Spherical & octahedral	25,60,100 °C	[77]
	*Spirulina platensis*	Extracellular	~12	Spherical	25	[78, 79]
**Microalgae**	*Chlamydomonas reinhardtii*	Intracellular & extracellular	5–15(in vitro), 5–35 (in vivo)	Round/rectangular	RT	[17]
	*Chlorella vulgaris*	Intracellular	~ 10	Spherical	28	[80, 81]
	*Nannochloropsis oculate*	Intracellular	~ 19	Spherical	28	[82]
**Macroalgae**	*Caulerpa racemose*	Extracellular	5–25	Spherical/triangular	RT	[83]
	*Codium capitatum*	–	~ 30		RT	[84]
	*Ulva fasciata*	Extracellular	28–41	Spherical	RT	[85]
	*Padina gymnospor*	–	25–40	Spherical	30	[86]
	*Padina pavonica*	Extracellular	45–64	Spherical	-	[87]
	*Gelidiella acerosa*	–	22	Spherical	RT	[23]
	*Gracilaria dura*	–	6	Spherical	25,60 & 100	[88]
	*Hypnea musciformis*	–	40–65	Spherical	RT	[89]

There are several different parameters that can alter the physical and chemical properties of NPs like shape, size, and stability. These parameters include temperature, pH, initial concentration and type of the metals, duration of the exposure, type and concentration of the reducing agents in the aqueous phase, etc. [90].

## Algae as one of the most appropriate agents for biological AgNP synthesis

These days, the use of algae for the biosynthesis of nanoparticles is prevalent. The use of algae is mainly due to their high capacity to take in metals and reduce metal ions, relatively low production costs, and most importantly their ability to produce nanoparticles at a large scale [91, 92]. Another interesting feature is their ability to tolerate harsh atmospheric conditions more effectively than other microorganisms [93]. Both live and dead dry biomass of algae can be used for nanoparticle biosynthesis, and hence, they are known as “Bionanofactories ”[94]. Another added advantage of using algae is the time required for the synthesis of silver nanoparticles. Alga-mediated synthesis requires less time compared to other microbes. The time required by *E. coli* to synthesize AgNPs was around 60 h [95] whereas *Caulerpa racemose* produced nanoparticles in just 3 h [83]. Silver nanoparticles synthesized by algae contain hydrophilic surface groups such as sulphate, carboxyl, and hydroxyl which gives them unique applicability. They can be used in medical treatment as algae themselves do not fabricate any toxic or harmful substance [15].

## Different methods of algal nanoparticle synthesis

**Fig. 3:**
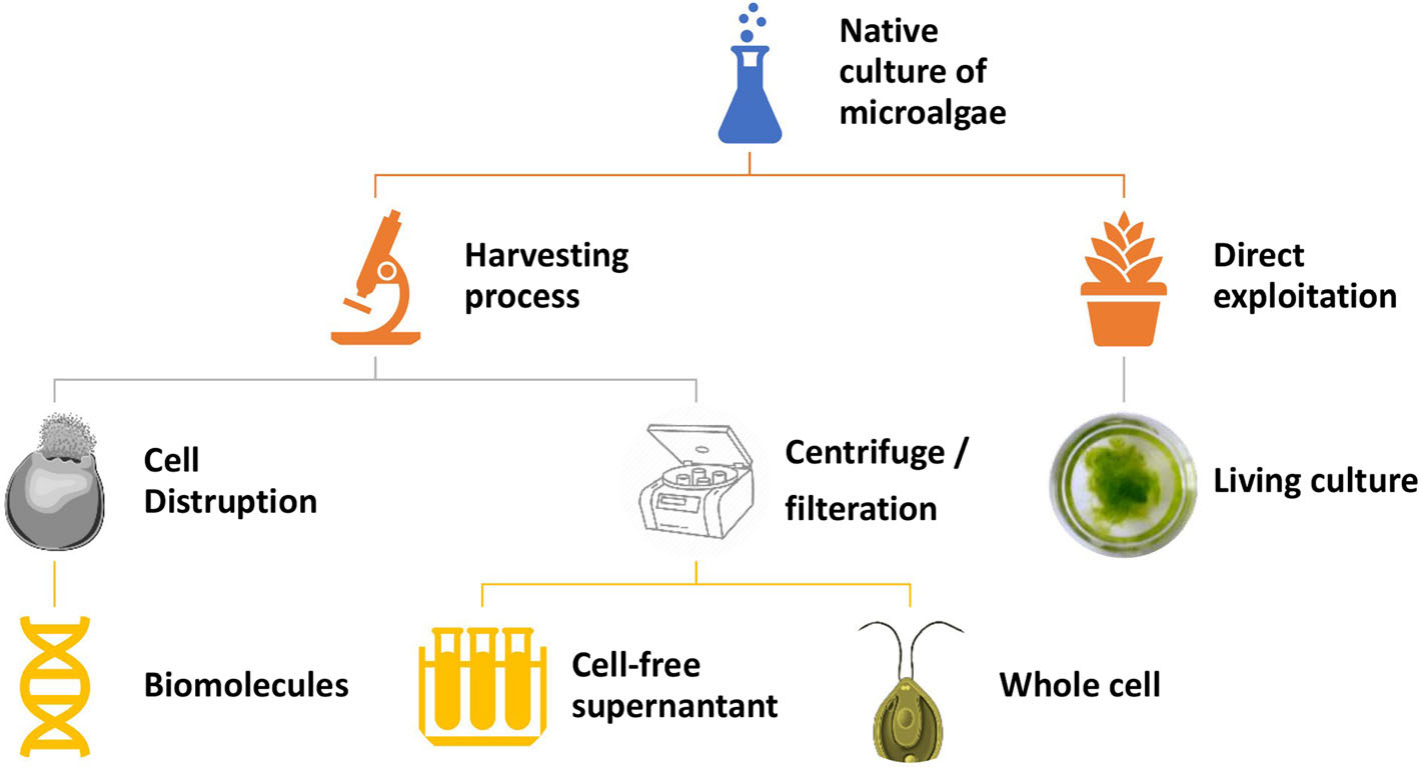
Different methodologies for the biosynthesis of silver nanoparticles using algae

Usually, an algal species synthesized NP by accumulating and subsequently reducing the cations. They can be synthesized from algal biomass using either the extracellular or intracellular mechanism (Fig. 3). There are many different approaches that can be employed for the biosynthesis of algal nanoparticles (Fig. 3).

The bio-reduction of a metal ion to its nanoparticle occurs on the surface of the algal cell in the extracellular pathway whereas in the intra-cellular mechanism the bio-reduction through enzymatic activity occurs inside the cell wall and cell membrane [74].

However, most studies related to the synthesis of silver nanoparticles with the algal biomass involve:

1. Preparation of algal extract by heating or boiling it in water or organic solvent for a particular time and at a specific temperature.
2. Molar solution preparation of ionic metallic compounds.
3. Incubation of algal solution and molar solutions of ionic metallic compounds for a particular period with either continuous stirring or without stirring in a regulated environment [90].

### Synthesis of nanoparticles using living culture

The use of living algal culture for the production of nanoparticles is one of the simplest methods. In this method, the live culture of algae is used without any additional processing steps. Evidence for the synthesis of Ag, Au nanoparticles using living cyanobacterial strains of *Anabaena flos-aquae*, *Calothrix pulvinata*, and *Leptolyngbya foveolarum* are already present. Synthesis can take place across two district pathways. The first one is the intracellular pathway in which the synthesis of nanoparticles occurs within the cell and the other pathway is the extracellular pathway in which the algae secret some bioactive compounds in the surrounding which facilitates the synthesis of nanoparticles. In algae, the extracellular pathway is more prominent than the intracellular pathway. There are numerous reports available in which researchers performed various experiments to synthesize silver nanoparticles using living culture [15]. In a study conducted by Mohseniazar et al., three different genera of microalga living cultures (*Nannochloropsis oculata* (Ochrophyta), *Dunaliella salina*, and *C. vulgaris* (Chlorophyta) were exposed to three different concentrations (1 mM, 2 mM, 5 mM) of aqueous AgNO_3_ solution. They observed that out of three algae, two species, *N. oculata* and *C. vulgaris*, were able to synthesize nanoparticles but only at 1 mM AgNO_3_ concentration [96].. The study indicates the synthesis of the nanoparticle depends highly on the algal species and the concentration of the AgNO_3_ solution used in the process. To ensure proper synthesis, optimization of culture conditions is mandatory.

In another experiment performed by Kadukova et al., researchers tried to evaluate the influence of two distinct parameters on the synthesis of silver nanoparticles by *Parachlorella kessleri*—initial silver concentration and culture age [97]. In this, AgNPs were produced by inoculating the algae in an aqueous solution of 0.5 mM AgNO_3_. Algal culture grown for 1, 2, 3, and 4 weeks on an agar plate was used to study the effect of culture age. The solution with an Ag+ ion concentration of 0.5, 1, and 2 mM was used for the study of the effect of initial silver concentration. UV-Vis spectroscopy was then used to confirm that the nanoparticles were present. They found that the age of culture has no effect on NP production but the change in initial silver concentration affects the synthesis of AgNP [97].

### Synthesis of silver nanoparticles using extracted biomolecules

Biomolecules are the organic molecules that are present inside the cells. They are mainly composed of carbon, nitrogen, oxygen, hydrogen, phosphorous, and sulphur. Different types of biomolecules include amino acids and proteins, carbohydrates, lipids, nucleic acid, and some small organic molecules [63]. There are several biomolecules reported to be associated with the algal synthesis of AgNP. Table 4 enlists different types of biomolecules, their source algae, and the physical property of AgNPs synthesized by them.

**Table 4:** List of biomolecules that are responsible for synthesizing silver nanoparticles

***Algae***	Biomolecule involved in AgNp synthesis	Size/shape	Reference
***C. reinhardtii***	Histone (H4)	~ 20 nm	[17]
***Chlorella vulgaris***	Polysaccharide (glucose, fructose, maltose, lactose, rhamnose, arabinose)	5.76 nm (average)/spherical	[98]
***Amphora*sp.**	Fucoxanthin	20–25 nm/spherical	[99]
***Navicula (Diatom)***	Fucoxanthin	70–80nm	[100]
***Limnothrix sp.***	C-Phycocyanin	25.65 ± 2 nm/spherical & elongated	[101]
***Spirulina*sp.**	C-Phycocyanin	13.85 ± 2 nm/spherical	[101]
***Laurencia obtusa***	Polysaccharides	4–10nm/spherical	[102]

**Fig. 4:**
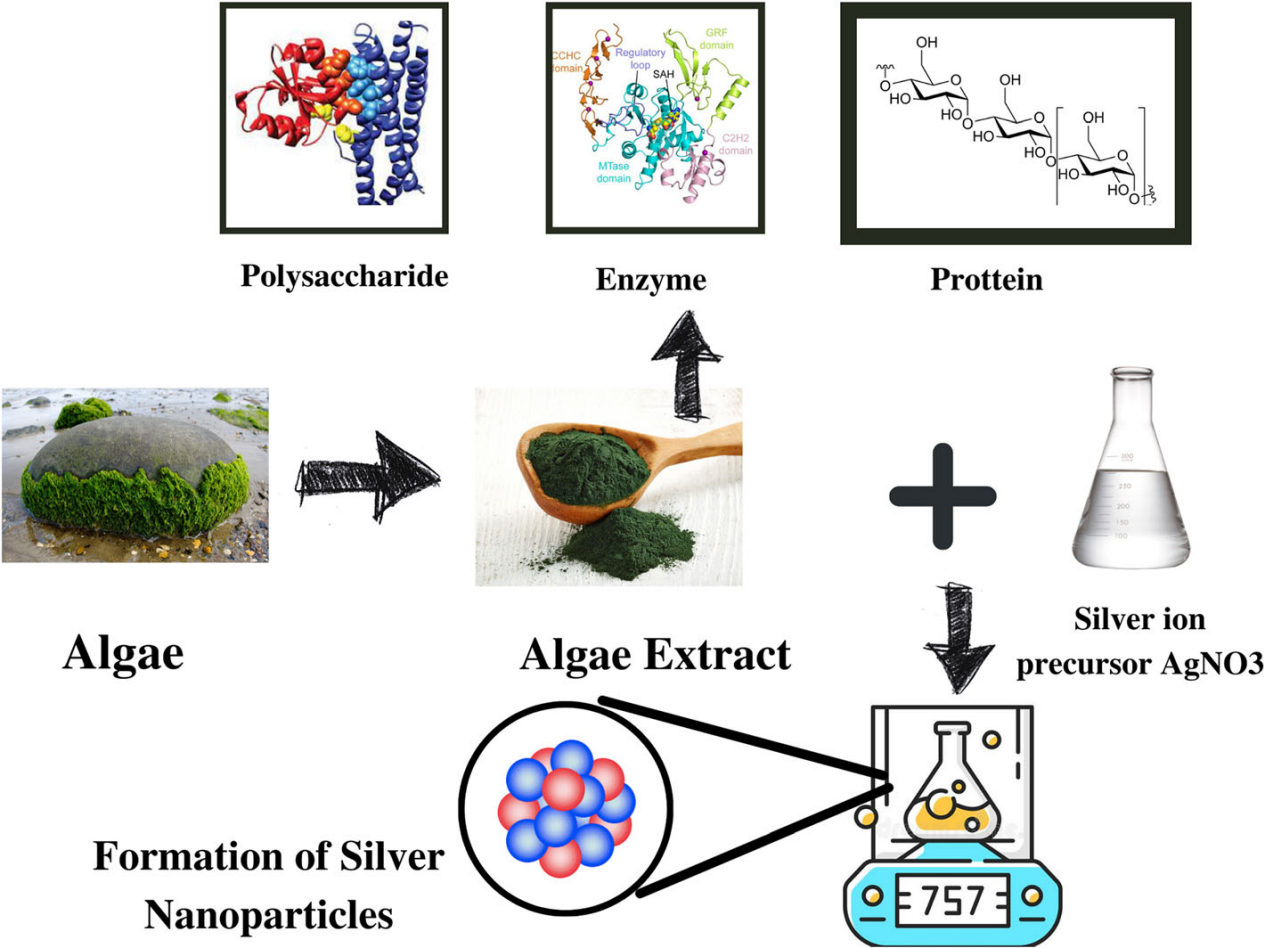
Mechanism of biosynthesis of NPs using algae using extracted biomolecule

For the extraction of the biomolecules, the cells need to be lysed. There are several methods for algal cell disruption including thermolysis, bead milling, ultrasonication, and laser treatment, grinding, etc. [103]. Once extracted from the cells, it is possible to use these biomolecules for synthesizing silver nanoparticles (Fig. 4).

Xie et al. reported the synthesis of silver nanoparticles with proteins extracted from algae (*Chlorella vulgaris*) [104]. For this, they used a 10-day-old culture of *C. vulgaris* and lyophilized it. After lyophilization, filtration was done through Whatman Autovials (0.45 μm) and proteins were separated by dialysis. The proteins that were of high molecular weight were collected and called algal proteins (aP). After that, proteins extracted were modified in four different ways to study their effect on the synthesis of nanoparticles using algae. These modifications were as follows: denaturation of aP, tyrosine (Tyr) residues in aP were chemically modified with the help of N-acetylimidazole (NAI), NAI-modified aP protein was deacetylated of by an inorganic compound hydroxylamine, amination of the carboxyl groups of aP to study their effect on biosynthesis or properties of nanoparticles. The algal extract (i.e proteins) was incubated with 1 mM silver nitrate. Because of surface plasmon resonance (SPR) of Ag nanocrystals in the visible region, the colour of the solution changed from light yellow to pinkish red. A transmission electron microscope (TEM) was performed to determine the shape of AgNPs. During TEM analysis, Ag nanocrystals of three different shapes, i.e. circular, triangular, and rod-like, were observed. This confirmed that proteins extracted were active biomolecules involved in the synthesis of Ag NP. SDS-PAGE (Sodium dodecyl sulphate-polyacrylamide gel electrophoresis) analysis showed that the molecular weight of proteins was in a range of 10–50 kDa. Through several different experiments, they also found that the hydroxyl group of tyrosine residue is responsible for Ag ion reduction. In addition, they found that the silver ion reduction kinetics often relies on moieties such as histidine and cysteine residues present in the proteins. Due to carboxyl groups in Asp and/or Glu residues, anisotropic growth of Ag nanocrystals to nanoplates happens. They also observed that the kinetics of Ag nanoplate formation was also dependent on the ratio of carboxyl groups to Tyr per peptide molecule [104].

In another experiment by Barwal et al. cellular proteins present in *C. reinhardtii* viz. histone (H4), carbonic anhydrase (CA), ferredoxin and ferredoxin NADP^+^ reductase (FNR), superoxide dismutase (SOD), sedoheptulose-1, 7-bisphosphatase (SBPase), ATP synthase, RuBP carboxylase, and oxygen-evolving enhancer protein (OEE) were associated with biosynthesized of AgNPs. They also found that protein-depleted fractions resulted in the alteration in size and rate of biosynthesis of AgNPs. These results confirmed the involvement of these proteins in *C. reinhardtii*-mediated biosynthesis of AgNPs [105].

Not only proteins even polysaccharides are reported to be involved in AgNP synthesis by algae. According to a report by El-Naggar et al., soluble polysaccharide extracted from *Chlorella vulgaris* can lead to biosynthesis of AgNPs. Soluble polysaccharide comprises monomeric units of glucose, rhamnose, maltose, lactose, fructose, and arabinose. They reported that the synthesis of NPs occurs after incubating the soluble polysaccharide with 100mM AgNO_3_ for 24 h in dark conditions. TEM analysis of AgNPs found that most of them were spherical with an average size of 5.76 nm [98].

Other than proteins and polysaccharides, the photosynthetic pigments are also responsible for reducing silver ions to silver nanoparticles. One such research was done by Jena et al., in which they used an aqueous extract of diatom *Amphora* sp. for pigment-mediated biogenic synthesis of silver nanoparticles. They noticed that there was a higher production of stable silver nanoparticles when the reaction mixture was exposed to light. It was discovered that the light-sensitive photosynthetic pigment fucoxanthin was primarily responsible for the reduction of Ag^+^ to Ag^0^. Spectroscopic studies have confirmed the development of silver nanoparticles as significant surface plasmon resonance was observed at 415 nm. The size of synthesized nanoparticles was around 20–25 nm and most of them were spherical [99].

### Synthesis of silver nanoparticles using cell-free supernatant

**Fig. 5:**
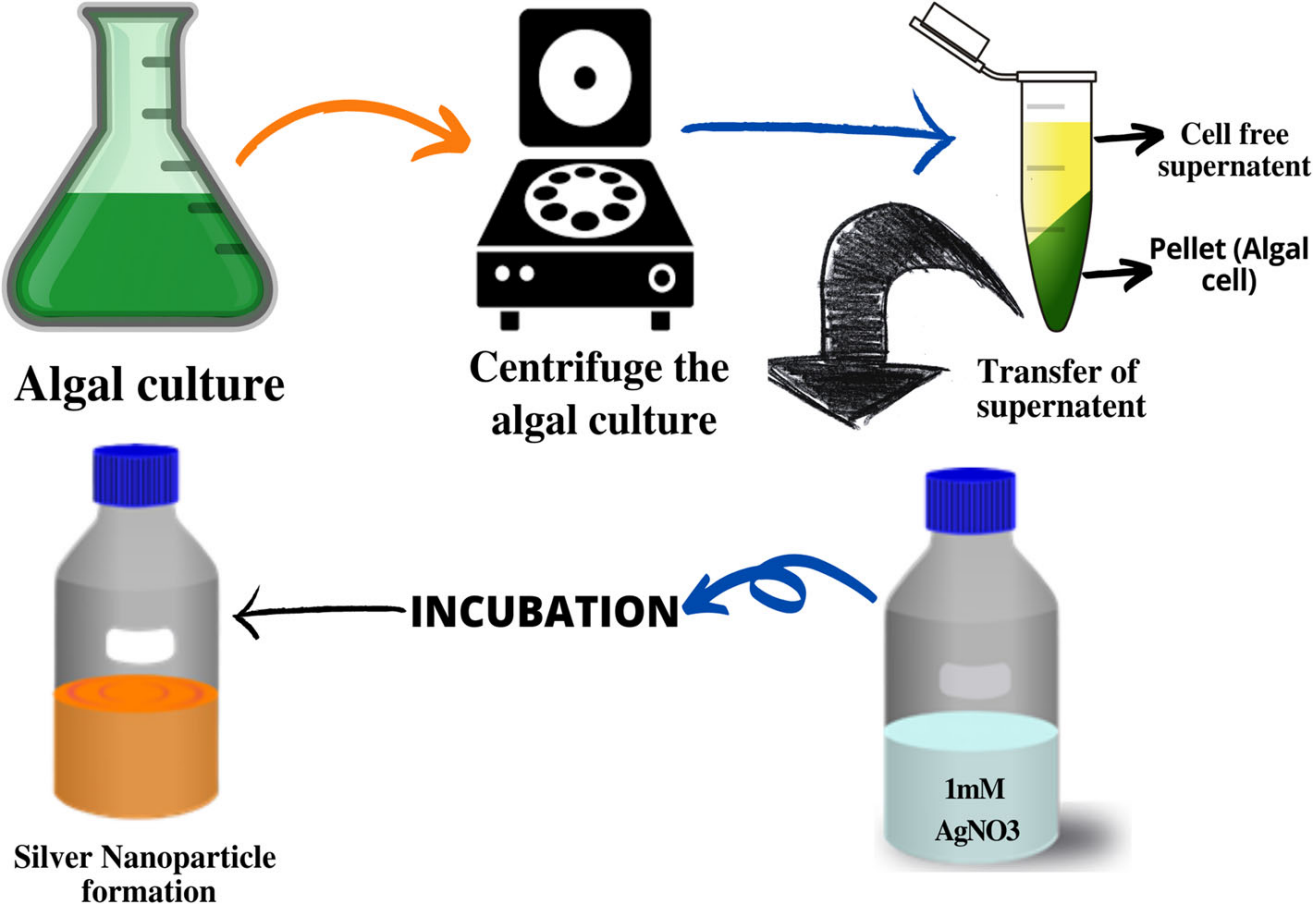
Mechanism for the synthesis of silver nanoparticles by the cell-free culture of algae

Unlike the previously mentioned methods like the use of live cells or biomolecules extracted from the algae, this method involves the use of cell-free extract. Cell-free extract can be obtained by growing the algae in a liquid culture media and then separating the biomass from the media by centrifugation or filtration. The supernatant obtained by this process can be used for the production of nanoparticles [15, 92] (Fig. 5).

In a research conducted by Sharma et al., biosynthesis of algal silver nanoparticles was carried out using a cell-free extract of *Spirulina platensis* [106]. For the preparation of the microalga extract, 5 g of *S. platensis* was suspended in 100 ml of distilled water for 15 min at 100° C, and then, after cooling, they centrifuged it at 10,000 rpm for 15 min and collected the supernatant. Then, for the synthesis of AgNP, 2 ml of the supernatant was added in 100 mL of 1mM AgNO_3_ solution and the flask was kept at 60 °C for 10 min. After 10 min, the colour of the solution changed from yellow to dark brown which indicates the reduction of Ag ions to Ag nanoparticles. Analysis of nanoparticles by TEM revealed that most of the synthesized nanoparticles were spherical and their size ranges between 30 and 50nm [106].

### Synthesis of silver nanoparticles using the whole cell

This process involves the removal of growth media from the algal culture by centrifugation, filtration, or simple washing of the cells. Followed by separation, the algal biomass is used for the synthesis, but before adding to the precursor salt solution, the cells are resuspended into distilled water [15]. The use of the whole cell for the synthesis of AgNP with the help of *C. vulgaris* was demonstrated by Soleimani and Habibi-Pirkoohi [80]. In this, the cells of *C. vulgaris* were centrifuged at 5000 rpm for 15 min at 4 °C. The cells obtained by discarding the supernatant were washed with distilled water and mixed with 200mM AgNO_3_ in order to get a final concentration of 100 mM. After 48 h, the green colour of *C. vulgaris* turned to brown which indicated the presence of silver nanoparticles. A surface plasmon resonance peak was observed in UV-vis spectroscopy at approximately 450 nm which verified the formation of silver nanoparticles. The synthesized nanoparticles were spherical in shape and were about 10 nm in size [80]. The whole-cell extract of *Plectonema boryanum*, *Euglena gracilis*, and *Euglena intermedia* (Euglenozoa) have already been reported for the synthesis of silver nanoparticles. However, the major disadvantage of this method is that the cell loses their metabolic activity within hours as they are isolated from their growth media and suspended in distilled water [107].

## Factors affecting silver nanoparticle synthesis by algae

**Fig. 6:**
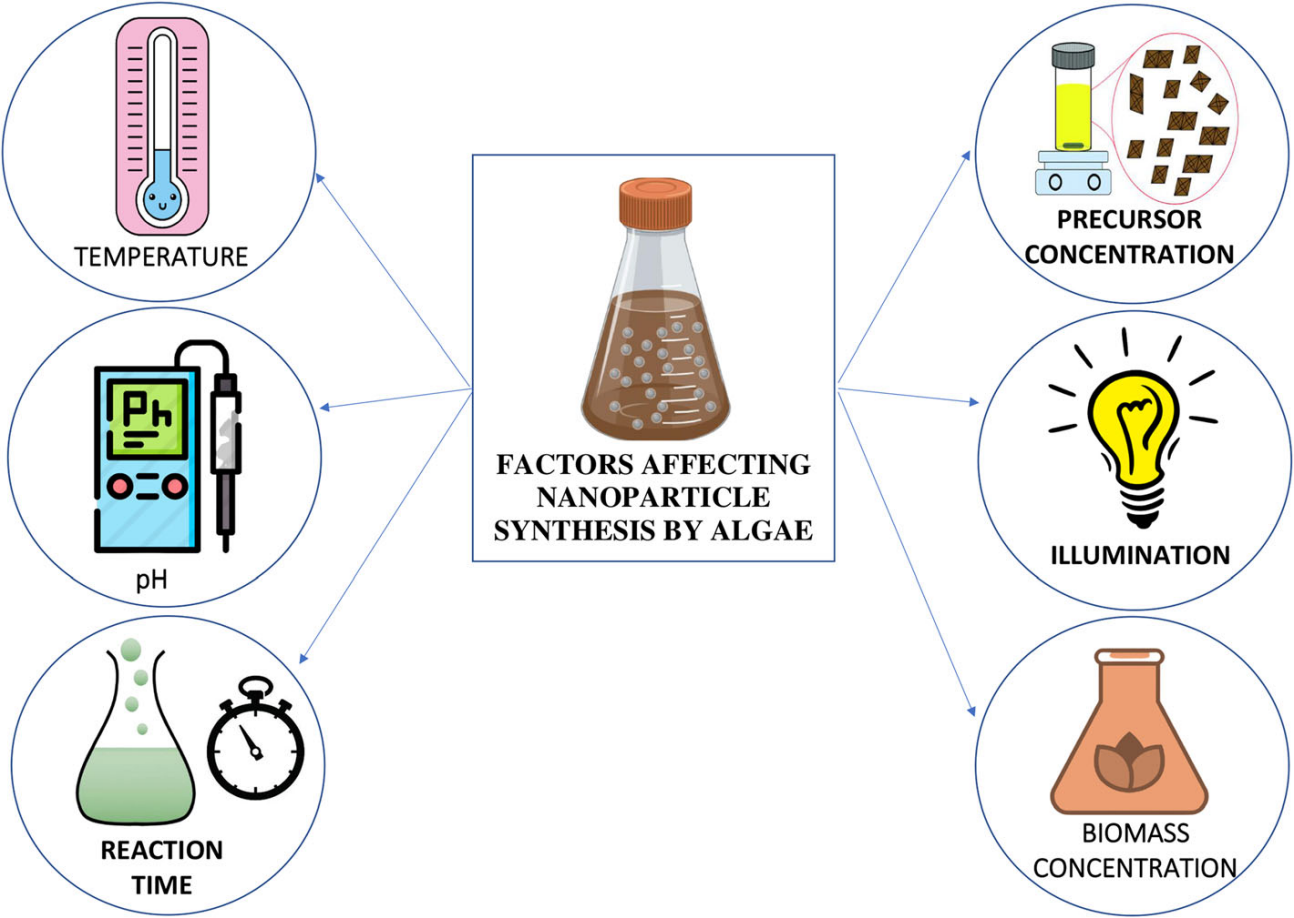
Factors affecting the synthesis of silver nanoparticles

Algal culture in different forms can be used very efficiently for nanoparticle synthesis, but for each of the synthesis process apart from the algae, certain other conditions have to be maintained properly. There are several different conditions like, physical, chemical, and environmental factors that affect the biosynthesis of silver nanoparticles with the help of algae. Some of the important factors are extract or biomass concentration, pH, incubation time, illumination, precursor concentration, and temperature (Fig. 6).

### Effect of extract concentration

The concentration of the algal extract is one of the most important factors that influence the biosynthesis of silver nanoparticles. It has been observed that biomass/extract concentration is directly related to the yield of nanoparticles. In addition, the size and structure of nanoparticles are also significantly influenced by the concentration of substrate [15]. Aboelfetoh et al. demonstrated the effect of *C. serrulata* extract concentration on the AgNP biosynthesis [108]. They observed that on increasing the concentration of the extract gradually (5 to 20%) in 10^−3^ M AgNO_3_, the intensity of SPR increased and there was a shift towards a lower wavelength (435 nm). This shift was due to a decrease in the mean size of AgNPs [108]. On further increasing the concentration to 25% there was a reduction in SPR intensity due to the aggregation of nanoparticles [108]. The study depicts that even though increasing the concentration can increase in production of AgNP, beyond a threshold concentration agglomeration happens. Agglomeration of nanoparticles can cause a reduction in their functional properties and economic importance. Hence for methods involving cellular extract, it is very important to standardize an optimum concentration along with other parameters.

### pH

The size and morphology of the biologically synthesized nanoparticles can be influenced greatly by the change in pH [92, 109]. It is because the electrical charge of biomass and capping agents are altered strongly at different pH conditions which causes alteration in their ability to bind and reduce metal ions. Hamouda et al. showed that the optimum pH for AgNP synthesis by *O. limnetica* was 6.7, but as soon as the pH was altered to 4.7 or 5.7, NP synthesis was completely inhibited [109]. Another group of researchers stated that a large number of functional groups are available for silver binding at a slightly acidic pH and this allows a large number of Ag ions to bind and form nanoparticles [110]. Studies related to change in shape and size of nanoparticles by altering the pH of the reaction medium have also been reported [111]. Several reports exhibit that an acidic medium promotes the production of large-size AgNPs whereas small-size AgNPs were formed in the alkaline medium [112].

### Temperature

Temperature is another important factor that influences the biosynthesis of silver nanoparticles. In general, the rate of reaction and production of NP increases with an increase in temperature [111]. Not only the rate of the reaction increases with the increase in temperature, but it can also help in higher production of NP and regulate the size of the particles too. With an increase in temperature, the size of nanoparticles also increases [113].

Alteration in temperature can also affect the shape of nanoparticles. Lengke et al. reported that during the synthesis of AgNP with cyanobacterium *Plectonema boryanum*, the shape of the AgNp changed with a temperature change. Spherical silver nanoparticles were observed at all temperatures ranging from 25 to 100 °C but a octahedral-shaped NP was produced only at 100 °C [77]. Prasad et al. in an experiment found that the average size of nanoparticles synthesized by the brown alga *Cystophora moniliformis* at a temperature less than 65 °C was 75 nm. He also reported that the aggregation of nanoparticles occurs at a higher temperature (85–90 °C) and a cluster of particles with size > 2 μm was observed at 95 °C [114]. Hence, there is a need for optimization of the temperature for AgNP production and the temperature condition is highly dependent on the algal stain and the method being used for the synthesis. This is interesting in another way because by controlling this parameter NP of different sizes can be produced easily.

### Incubation time/reaction time

Biological synthesis of NP involves incubation of the AgNO_3_ solution with the microorganisms or their extract for a fixed duration used certain physical and chemical conditions. The biosynthesis of silver nanoparticles by algae also depends on the reaction time or incubation time. In general, with an increase in reaction time, the number of nanoparticles increases but only for a certain amount of time. After that, there might be an agglomeration of silver nanoparticles due to their instability. However, if the nanoparticles synthesized are stable then there will be no effect of increasing time on them [115]. The effect of incubation time also depends on the organism that is being used for the synthesis. Kannan et al. observed that *Codium capitatum* took around 48 h for the production of nanoparticles from the precursor whereas it took just 30 min for synthesis by *Chaetomorpha linum* [84, 116]*.* Exposure time can also influence the size of particles.

### Precursor concentration

It is well known that precursor concentration not only influences the yield but also affects the morphology of synthesized nanoparticles. In a study conducted by Rahman et al., the researchers used two different precursor concentrations (0.650 mM and 1.250 mM) for the synthesis of silver nanoparticles using *C. reinhardtii*. They reported that the formation of AgNPs was faster at 0.650 mM of AgNO_3_ than at 1.250 mM. This depicts that the rate of conversion of Ag^+^ to AgNps depends on the concentration of precursors [117]. It has also been observed that precursor concentration directly affects the number of nanoparticles synthesized, i.e. the higher the concentration, the greater the yield. However, the viability of cells gets reduced with higher yield due to the cytotoxic effect of both NPs and cations and therefore changes the yield and morphology of synthesized NP [15]. The size of nanoparticles can also be affected by the precursor concentration. With an increase in precursor concentration, the size of NPs also increases [118].

### Illumination

Light can act as a catalyst for many reactions. Illumination is a critical physical factor that can affect the synthesis of AgNP. There are multiple experimental evidence that establish the effect of illumination during nanoparticle synthesis. Patel et al. in an experiment showed that extracellular polysaccharides isolated from the *Scenedesmus* sp. were able to synthesize silver nanoparticles in the presence of light but failed to do so in the dark. This indicates that how important light is for the synthesis of silver nanoparticles in this condition [101].. Another group of researchers led by Bao et al. reported the same observation by using an aqueous cell extract of *Neochloris oleoabundans*. They found that the biosynthesis of AgNPs needs illumination of white, blue, or purple light. The same extract failed to show NP synthesis when the reaction was carried out in dark conditions or in the presence of orange or red light [119]. The study clearly indicates that it is not only illumination; there are particular wavelengths that assist in the AgNP synthesis by different methods. Even UV light can affect the size of AgNPs [101].

In a study by El-Naggar et al. on phycocyanin mediated synthesis of AgNP it was reported that the even in presence of light, synthesis of AgNP was impossible if the pigment phycocyanin was omitted from the reaction mixture. They concluded that the chromophores on being exposed to light get excited, and at that condition, they cause a reduction of the AgNO_3_ in the medium to form AgNP [34].

In a current study by Rahaman et al., it was established that the synthesis of AgNP by extracellular polymeric substances (EPS) of *Chlamydomonas reinhardtii* is affected by both the EPS concentration and light intensity [120]. The absorption of Ag^+^ was carried out by the EPS molecules but the conversion of Ag^+^ to AgNP was driven by light. With the progress in the reaction condition, the concentration of H^+^ increased thereby causing a decrease in the pH. It was observed that with an increase in the intensity of the light the decrease in pH and concentration of Ag^+^ also decreased. This observation indicated the involvement of the light in regulating the AgNP production with EPS [120]. Hence, under certain reaction conditions, illumination can assist in the formation of AgNP by causing a reduction of cations of the metal M^n+^ to M^0^; however, the wavelength and the intensity are highly dependent on the components of the system.

### Capping agent

During the bottom-up approach of nanoparticle synthesis, nucleation and growth of nanoparticles occur. The first step of the synthesis, known as the reduction reaction, involves the reduction of metal ion precursors with the help of a reducing agent. This induces the nucleation of the atoms and then the growth of the nuclei (seeds) into metal nanoparticles. There are different theories for the nucleation and growth mechanisms of nanoparticles such as the LaMer mechanism, Ostwald ripening, Finke-Watzky mechanism, coalescence, and oriented attachment, and intra-particle growth. The silver nanoparticle synthesis mostly involves two mechanisms: Ostwald ripening and Coalescence [121]. Ostwald ripening is a thermodynamically driven process in which small nanoparticles are dissolved and are re-deposited onto larger nanoparticles [122]. Due to the high solubility and surface energy of small particles, they dissolve and larger particles grow even more**.** Coalescence is the attachment of the smaller particles through a random orientation of their lattices to produce larger particles and thereby reducing the number of particles within the solution [121].

**Fig. 7:**
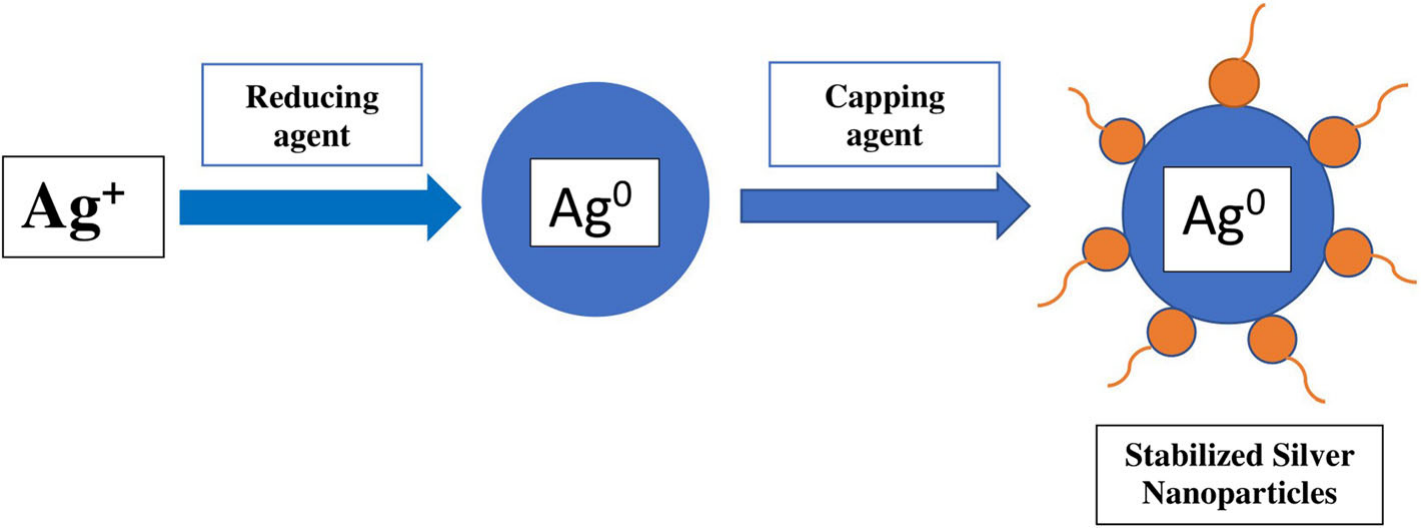
The action of capping agent on the Ag NP

Capping agents are amphiphilic molecules that possess a polar head and a non-polar tail region. The polar head group coordinates to the metal atom of the nanocrystals that are formed by agglomeration, whereas the tail region interacts with the surrounding medium [123]. (Fig. 7) Capping agents generally used are ionic and non-ionic surfactants and polymers. In bottom-up approaches, the different forces such as Van der Waals interaction, capillary interaction, surface tension, and hydrophobic interaction, and H-bonding effect mediates the self-assembly of nanoparticles. By exploiting these mechanisms using different capping agents, stable metal nanoparticles can be synthesized [124]. The stability, shape, and size of the nanoparticles determine their applications in different fields. Agglomerated silver nanoparticles may not be desired for certain applications like biomedicine, nanosensors, etc. Different capping agents can be utilized to obtain the desired shape and size of AgNPs. Some capping agents have dual roles in reducing and capping the nanoparticles.

#### Capping agent in green synthesis of silver nanoparticles

In a study performed by Jayaprakash et al., Silver nanospheres (AgNS) capped with Triton X-100 were synthesized by the microwave-assisted method [125]. There was a dual reduction and capping role for Triton X-100 in the presence of microwave radiations. HR-TEM (High-resolution transmission electron microscopy) revealed the spherical morphology of the AgNS with an average diameter of 5 nm. As stated in previous literature, the Raman spectrum showed different bands due to Triton X-100 and another additional band at 1608 cm^−1^ was seen due to the C=O stretching vibration in the aldehyde group, which clearly shows that the -OH group in TX-100 has oxidized through the reduction of Ag+ ions to the C=O group. In addition, compared to the Raman bands produced by pure Triton X-100, all the bands observed were shifted to lower wavelengths [125]. In another experiment, Tollens’ reagent was used for NP synthesis along with microwave irradiation. The process reported the use of a biopolymer xylan as a capping agent; however, as observed by the researchers, xylan functioned as a reducing agent too.

The diameter of the Ag NPs synthesized by this process was in the range of 20–35 nm [126].

Plant-mediated synthesis of AgNPs using aqueous extracts of fresh leaves of *Impatiens balsamina and Lantana camara* as reducing agents was performed and the average size of the AgNPs obtained was less than 24 nm which was determined using TEM. TEM images revealed the presence of an organic layer around the AgNPs. This layer was due to the presence of polyphenolic compounds like flavonoids and terpenoids which facilitated the reduction of Ag^+^ as well as the capping of the AgNPs [127]. *Aloe vera* extract was also reported as a reducing and capping agent for the biosynthesis of Ag NPs under microwave irradiation. Ammoniacal silver nitrate solution was used as the metal ion precursor, shape and size of the synthesized NPs were studied by TEM and SEM (Scanning Electron Microscope) imaging. The NPs produced by this method were octahedral in shape and 5–50 nm in size. The FTIR (Fourier Transform Infrared Spectroscopy) spectrum for the NPs showed a few peaks which indicated the presence of flavanones and terpenoids on the surface of AgNPs as capping agents [128]. Many such studies have proved that the biomolecules present in the plant extracts play the roles of reducing agent and capping agent. Extracellular biosynthesis of AgNPs was carried out using the aqueous culture filtrate of the endophytic fungus *Pestalotiopsis microspore*. The TEM images showed spherical AgNPs of diameter 2–10 nm and the FTIR spectrum indicated the presence of phenolic compounds (flavonoids and triterpenoids) and proteins in the fungal culture filtrate which stabilized the AgNPs by acting as capping agents [129]. In another study, the culture filtrate of a filamentous fungus called *Fusarium oxysporum* was used for the extracellular biosynthesis of AgNPs. The size of the AgNPs was in the range 21.3–37.3 nm and the spherical shape. The FTIR spectrum of these NPs showed the presence of amide I and amide II in the fungal extract which was the capping agent [130].

The biosynthesis of AgNPs by a new strain of *Pseudomonas* was performed and spherical polydispersed AgNPs of average size 50 nm were obtained. However, in this case, the presence of capping proteins on the surface of AgNPs produced by the bacteria was confirmed by the FTIR study. The interactions that stabilized the NP were mostly between the free amine groups and cysteine residues of the protein and the NP [131]**.** *Bacillus subtilis* was used in another study for the biosynthesis of AgNPs and spherical-shaped AgNPs of 3 to 20 nm were obtained. The FTIR spectrum showed some peaks which indicated the existence of proteins that acted as capping agents [132]**.**

Different alga-mediated synthesis of AgNP is well established now. A study involving the use of *Chlamydomonas reinhardtii* exhibited the involvement of cellar proteins as capping agents. Intracellular synthesis of AgNP was carried out which led to the formation of rounded/rectangular AgNPs of size 5–15 nm and 5–35 nm respectively. The TEM and SEM images showed bright spots inside the algal cells due to the synthesis of AgNPs and it was observed that the AgNPs were localized in the periplasm and cytoplasm and the basal bodies of the flagella. MALDI-MS MS (Matrix-assisted laser desorption ionization-tandem mass spectrometry) confirmed the function of cellular proteins in biosynthesis and capping. Most of the AgNP-associated proteins were involved in the oxidoreductive mechanism in the algal cells, ATPase activity, sedoheptulose-1,7-bisphosphatase, carbonic anhydrase, FNR, SOD, oxygen-evolving enhancer protein, ribulose bisphosphate carboxylase, and nuclear histone (H4). The majority of these proteins acted as reducing as well as capping agents [17]. In another study, the biosynthesis of AgNPs by the freshwater green algae *Pithophora oedogonia* was carried out and the results of the SEM and DLS (dynamic light scattering) showed the formation of cubic and hexagonal AgNPs of 25–44-nm size. The FTIR spectrum showed certain peaks corresponding to the presence of terpenoids, long-chain fatty acids, and secondary amide derivatives which capped the AgNPs and stabilized them [133]**.**

Biogenic synthesis of AgNPs was also carried by cell-free extract of *Saccharomyces boulardii*. TEM images showed the presence of monodispersed spherical AgNPs with the size range of 3–10 nm. The FTIR results showed certain bands that corresponded to the involvement of proteins in the reduction and stabilization/capping of the AgNPs. Further, the UV-Visible spectra of the cell-free extract showed strong absorption at 280 nm which may be due to the aromatic amino acid residues (tyrosine and tryptophan) present in the proteins. To confirm the role of proteins in the biosynthesis of stabilization of AgNPs, the yeast cells were subjected to heat treatment at various stages of growth. The heat-killed yeast cells were unable to synthesize AgNPs, this was probably due to the denaturation of the proteins by heat treatment [134].**.** Baker’s yeast (*Saccharomyces cerevisiae*) was used for the extracellular synthesis of AgNPs. Oval-shaped AgNPs of average size ~ 16.07 nm were obtained which was determined using XRD (X-ray diffraction). The FTIR revealed notable peaks which showed the capping of AgNPs by active biomolecules, alcohols, phenols, carboxylic acids and aromatic amines. These capping agents lead to effective stabilization of the AgNPs [135]. Table 5 shows other different capping agents in chemical and biological synthesis.

**Table 5:** List of different capping agents in chemical and biological syntheses

Method	Silver precursor	Reducing agent	Stabilizing/capping agents	Size (nm) & shape	Reference
**Chemical reduction**	AgNO_3_	Trisodium citrate	NaBH_4_	60 & 100 nm (0.75 ml and 1 ml of NaBH_4_ respectively) Spherical	[136]
**Chemical reduction**	AgNO_3_	Sodium citrate	NaBH_4_	~ 23 Spherical	[137]
**Chemical reduction**	AgNO_3_	MSA (mercaptosuccinic acid)	MSA	~ 65 Spherical	[137]
**Chemical reduction**	AgNO_3_	NaBH_4_	Thioctic acid	~ 44 Spherical	[137]
**Chemical reduction**	AgNO_3_	–	PVP	15 Spherical	[138]
**Chemical reduction**	AgNO_3_	–	Citrate	12.6 Spherical	[138]
**Chemical reduction**	AgNO_3_	–	BPEI (branched polyethyleneimine)	11.5 Spherical	[138]
**Chemical reduction**	AgNO_3_	Trisodium citrate	SDS (sodium dodecyl sulphate)	19 Spherical	[139]
**Chemical reduction**	AgNO_3_	Trisodium citrate	PEG	50 Spherical	[139]
**By plant**	AgNO_3_	*Cleome viscosa*	Alkaloids, phenolic compounds, tannins, proteins	5–30 Spherical	[140]
**Plant**	AgNO_3_	*Callicarpa maingayi*	Alkaloids, flavonoids, -OH, -NH, -C=O groups	13–15.6 Spherical	[141]
**Plant**	AgNO_3_	*Citrus limon*	Citric acid	16–18 Spherical	[142]
**Plant**	AgNO_3_	*Murraya koenigii*	Carboxylic acids, alcohol, ether, proteins	5–20 Spherical	[143]
**Fungi**	AgNO_3_	*Fusarium oxysporum*	Proteins	10–20 Spherical	[66]
**Fungi**	AgNO_3_	*Aspergillus oryzae*	Proteins	< 105 (depending on pH, temp, and conc. of AgNO_3_) Spherical	[67]
**Fungi**	AgNO_3_	*Trichoderma reesei*	Proteins	5–50 Spherical	[68]
**Bacteria**	AgNO_3_	*E.Coli*	-NH, -OH, Carbonyl, CN triple bond in proteins	118 Spherical	[144]
**Bacteria**	AgNO_3_	*Bacillus cereus*	Proteins	10–30 Spherical	[69]
**Brown algae**	AgNO_3_	*Padina* sp.	Polyphenols, Fatty acids (Hexadecanoic acid)	25–61.4 Spherical	[145]
**Marine red algae**	AgNO_3_	*Spyridia fusiforms*	Proteins, Secondary cyclic alcohols	5–50 Mostly spherical, some triangular and rounded rectangular	[146]

#### Advantages of using external capping agent

As we have seen that nanoparticles are of great use and have different applications in the field of medicines, pharmaceuticals, cosmetics, electronics, etc. but they can still be hazardous to the environment depending on the approach of synthesis [147]. With time, AgNPs tend to form aggregates and these aggregates are sometimes toxic to the environment. So, the stabilization of silver nanoparticles is an important issue. Stabilization can also be carried out by using external capping agents or surface modifiers [148]. The selection of capping agents should be done carefully as it will determine several different physical and chemical properties of the nanoparticles. While selecting a capping agent, one should keep in mind that the substance should be non-toxic, biodegradable, well dispersed, bio soluble, and biocompatible [147]. Sodium Dodecyl Sulphate (SDS), a commonly used capping agent is capable of reducing the toxicity of nanoparticle s[149]. Hamouda et al. compared the antimicrobial activity of silver nanoparticles with and without capping agent (SDS) and found that silver nanoparticles along with SDS have high antimicrobial activity than nanoparticles synthesized without capping agent [150]. The use of an external capping agent along with the biological method of synthesis can help in producing AgNP with desired properties.

## Conclusion

There is a wide application of Ag-NPs in the field of medicines, cosmetics, biosensors, therapeutics, etc. In the last few years, several attempts have been made to develop new methods of green synthesis. Green synthesis mostly involves the use of microbes or plants and hence its environment-friendly and sustainable approach towards nanoparticle synthesis. This review article highlights the wide application of AgNPs and their different modes of synthesis. Among all the living microorganisms, the synthesis of AgNPs by algae is the most popular because of their cost-effective method of production and extraction. Alga-mediated synthesis can be carried out using cell extract, cell-free extract, and living cells. Most of these methods involve the participation of biomolecules as capping agents for the synthesis. Despite having several advantages, the production of silver nanoparticles using algae is still limited due to the lack of information about the exact mechanism of synthesis. Further analysis is required to resolve the problems related to kinetics, yield, and cell viability to construct large-scale bioreactors for commercial-level production. In addition, extensive research is also required to recognize and evaluate the role of different biomolecules as reducing and capping agents.

However, if the process can be coupled with the use of external capping agents, then the stability of the NPs can be under manual regulation. This will probably allow obtaining NPs of higher efficiency. A regulated and qualitative synthesis of algae-mediated NPs can be accomplished with new emerging technologies, which will help to improve the characteristics and functionality of these nanoparticles for their industrial application.

## Data Availability

Not applicable.

## Abbreviations

aP: Algal proteins
CA: Carbonic anhydrase
CTAB: Cetyltrimethylammonium bromide
FNR: Ferredoxin NADP^+^ reductase
FTIR: Fourier transform infrared spectroscopy
HR-TEM: High-Resolution transmission electron microscopy
MALDI-MS MS: Matrix-assisted laser desorption ionization-tandem mass spectrometry
MRSA: Methicillin-resistant *Staphylococcus aureus*
DMF: N, N-dimethylformamide
NAI: N-Acetylimidazole
NP: Nanoparticles
OEE: Oxygen-evolving enhancer protein
PEG: Polyethylene glycol
PVA: Polyvinyl alcohol
SDS-PAGE: Sodium dodecyl sulphate-polyacrylamide gel electrophoresis
SBPase: Sedoheptulose-1, 7-bisphosphatase
AgNPs: Silver nanoparticles
AgNO_3_: Silver Nitrate
AgNS: Silver nanospheres
SOD: Superoxide dismutase
SPR: Surface plasmon resonance
TEM: Transmission electron microscope
XRD: X-ray diffraction
